# New Therapeutic Approaches in Obesity and Metabolic Syndrome Associated with Polycystic Ovary Syndrome

**DOI:** 10.7759/cureus.1844

**Published:** 2017-11-13

**Authors:** Fatima Saleem, Syed W Rizvi

**Affiliations:** 1 Internal medicine, King Edward Medical University Lahore, Pakistan; 2 R Endocrinology, New Jersey, Asst. Professor, Internal Medicine and Endocrinology, Umdnj

**Keywords:** polycystic ovary syndrome, obesity, metabolic syndrome, hyperandrogenism, metformin, inositols, insulin resistance, pcos

## Abstract

Polycystic ovary syndrome (PCOS) is a pathophysiological disorder affecting reproductive and metabolic indices. PCOS is commonly associated with a high prevalence of insulin resistance and obesity; this association carries an increased risk of developing metabolic syndrome, type 2 diabetes mellitus (T2DM), and cardiovascular disease. Guidelines recommend lifestyle modification, metformin, hormonal contraceptives (HCs), and bariatric surgery as the main treatment options in obese patients with PCOS. Studies are being conducted to test the efficacy of existing treatment options as well as to discover new therapies. This review focuses on the most recent advances in this regard and highlights new hypotheses and emerging studies to give a picture of the latest therapeutic trends in the treatment of obese patients with PCOS. In this respect, much attention is given to the role of inositols, the mediators of insulin action. A deficiency of d-chiro-inositol containing inositol-phospho-glycans may be the basis of insulin resistance frequently seen in PCOS patients. Moreover, evidence suggests the use of statins in obese women with PCOS, but guidelines call for further research. Adiponectin, quercetin, vitamin D, and anti-obesity drugs have also been studied and seem to have a useful role in the treatment of obesity and metabolic syndrome in PCOS. Many trials have been conducted on the use of non-pharmacological therapies. Therapies including resveratrol, acupuncture, and berberine have favorable effects in overweight PCOS patients. However, more research is needed to reveal the clinical complexity of PCOS and develop more effective treatment options.

## Introduction and background

Polycystic ovary syndrome (PCOS) is a complex disorder of ovarian dysfunction, but it may seem to be a relatively simple clinical condition. The Endocrine Society’s clinical practice guidelines define PCOS (using the Rotterdam criteria) as the presence of two or more of the following: hyperandrogenism, ovulatory dysfunction, or polycystic ovaries, whereas the disorders that mimic the clinical features of PCOS are excluded [1-2]. These disorders include hyperprolactinemia, thyroid disease (hypothyroidism and hyperthyroidism), and non-classic congenital adrenal hyperplasia [1]. There may be other forms of PCOS without showing a clear evidence of hyperandrogenism, but more data is needed to validate this supposition [2]. Also, depending on the presentation, other disorders such as acromegaly, androgen-secreting tumors, primary ovarian insufficiency, and Cushing’s syndrome should be excluded before confirming a PCOS diagnosis [1]. Moreover, hyperandrogenism is central to the presentation in adolescents, while there may be no consistent phenotype in postmenopausal women [1].

The Androgen Excess PCOS Society Task Force also suggests that PCOS should first be considered as a disorder of androgen excess or hyperandrogenism and that the most reliable indicator of hyperandrogenism is hirsutism and free testosterone levels [2]. The task force recognized that almost 70% to 90% of women with PCOS show a polycystic ovarian morphology on ultrasound, although the false positive rate is high [2]. To understand the scale of the problem and the importance of research and resource allocation for women with PCOS, a study was conducted in Australia [3]. It concludes that women with PCOS are associated with more non-obstetric and non-injury related hospital admissions as compared to women without PCOS. They also have a higher rate of admissions for menorrhagia, infertility, and miscarriage. They are more likely to require in vitro fertilization [3].

PCOS is commonly associated with a high prevalence of hyperinsulinemia, insulin resistance, and obesity. These patients are at an increased risk of metabolic syndrome, type 2 diabetes mellitus (T2DM), cardiovascular disease, and unopposed estrogen effects on the endometrium [2]. Moreover, an increased luteinizing hormone (LH) and luteinizing hormone to follicular stimulating hormone ratio (LH/FSH) is also observed [2]. This LH/FSH ratio value is much dependent on the assay used to measure the hormones. These patients should be evaluated for the risk of developing metabolic syndrome and its components including T2DM, hypertension, and hyperlipidemia [4].

The rate at which normal glucose tolerance progresses to impaired glucose tolerance leading to T2DM can be as high as 5% to 15% in three years [4]. This can increase the risk of acute myocardial infarction and stroke in overweight women with PCOS [4]. Obesity and PCOS have many common features in their pathogenesis. Both the disorders are linked to insulin resistance and hyperinsulinemia [5]. To better understand the pathogenesis and to show which condition comes first, more research is needed [5]. Lifestyle changes, pharmacological therapy, and bariatric surgery are the main interventions in managing PCOS with obesity and preventing metabolic comorbidities.

## Review

Lifestyle modification

The initial therapy for PCOS is weight loss. The importance of weight loss can be illustrated by the fact that a weight reduction of as little as 5% can restore regular menstruation and improve response to ovulation induction and fertility treatment [4]. The Endocrine Society clinical practice guidelines suggest the use of exercise therapy along with diet modification as the first-line treatment to manage obesity in adolescents and women with PCOS [1]. Regarding dietary intervention, thus far, no caloric restricted diet has been recommended in the guidelines. Early initiation of exercise alone, or in combination with dietary energy restrictions, helps in improving reproductive and metabolic indicators in women with PCOS and obesity [1,6]. Also, weight loss alone is insufficient in treating PCOS in women with normal BMI [1].

The molecular mechanism underlying the efficacy of lifestyle interventions and its effects was recently explained in a rodent model study [6]. It appears to be mediated through the normalization of the hypothalamic neuropeptides (cocaine and amphetamine-regulated transcript (CART) and Kisspeptin), and plasma sex-hormone binding globulin (SHBG) [6]. These findings suggest that early intervention with dietary restrictions and exercise in young adolescents with PCOS as well as in pre-pubertal children at risk of PCOS may improve metabolic, reproductive, and endocrine parameters. This improvement is caused by regulation of the neuropeptides in the hypothalamic-pituitary-gonadal axis [6]. Pretreatment weight loss is also imperative for infertility treatment in PCOS. Pretreatment lifestyle modification and weight loss (16 weeks), with or without concurrent oral contraceptive therapy, is associated with a significant improvement in the rate of ovulation and an even greater increase in live birth rates as compared to immediate fertility treatment without lifestyle modification [7].

Metformin

Among the pharmacological therapies to treat PCOS, insulin-sensitizing agents are fundamental to correct the underlying metabolic abnormalities. Both metformin and hormonal contraceptives (HCs) are the treatment of choice in adolescents with PCOS [1]. Metformin, the primary insulin-sensitizing agent, is commonly used alone or in a low-dose combination with flutamide, or with both flutamide and pioglitazone (PioFluMet) [8]. PCOS is linked to insulin resistance which results in a compensatory increase in insulin secretion by the islet cells of the pancreas to maintain normal glucose homeostasis [2]. This secondary hyperinsulinemia leads to many PCOS phenotypic features, such as ovarian hyperandrogenism and acanthosis nigricans. It decreases the production of SHBG. Insulin resistance further leads to metabolic complications including T2DM, dyslipidemia, and metabolic syndrome. Moreover, obesity in PCOS further worsens the metabolic parameters in PCOS women [2]. HCs are recommended as the first-line therapy for PCOS [1]. The guidelines recommend metformin in those patients with PCOS who have T2DM or impaired glucose tolerance and failed lifestyle modification. For women with PCOS with menstrual irregularity who cannot take or do not tolerate HCs, metformin is suggested as a second-line therapy [1].

Metformin appears to have a direct effect on steroidogenesis in the ovaries regardless of its influence on insulin sensitivity. Metformin also decreases LH-stimulated testosterone secretion soon after use [9]. However, the exact mechanism of action in PCOS is still not completely understood. Kurzthaler found no effect of metformin on body weight, insulin sensitivity and metabolic indices such as low-density lipoproteins (LDL), high-density lipoproteins (HDL), and triglycerides following three months of use. However, a significant decline in serum testosterone was observed after a relatively short course of treatment [9]. Likewise, when adolescents with hyperandrogenic type 1 diabetes mellitus (T1DM) were treated with metformin, serum androgens decreased significantly, but other parameters of PCOS like hirsutism, ovulation, and metabolic indices did not alter significantly [10]. Therefore, until now, metformin does not seem to have a beneficial role in treating hirsutism, acne, and infertility [1]. The Endocrine Society clinical practice guidelines suggest against the use of metformin as a first-line treatment of skin manifestations, hirsutism, for the prevention of pregnancy complications, or for the treatment of obesity [1].

However, Naderpoor, et al. recently proposed that metformin treatment considerably improves body mass index (BMI), serum lipids, and glucose homeostasis. A combination of lifestyle and metformin is more beneficial than lifestyle alone in reducing BMI and adipose tissue levels. It also regulates the menstrual cycle in women with PCOS [11]. Many of the existing studies on the role of metformin in PCOS have limitations based on small sample sizes, bias risk, and short durations [11].

Pioglitazone

Insulin-sensitizing agents are the initial treatment option in PCOS and pioglitazone is one of them. Treatment with pioglitazone is known to improve menstrual irregularities and hirsutism in PCOS. It also modifies other parameters linked to a higher risk of T2DM and cardiovascular diseases. Pioglitazone 30mg/day leads to an increase in adiponectin with a reduction of insulin resistance. Fibrinogen and C-reactive protein (CRP) levels are also decreased [12]. Pioglitazone significantly improves endocrine and metabolic parameters in women with PCOS, and it can be a suitable alternative to metformin in women who are unable to take or tolerate metformin [13]. However, its use has been limited to patients with diabetes because of the associated cancer risk of thiazolidinediones and the eventual weight gain. The international guidelines do not favor thiazolidinediones because they do not have an overall favorable risk-benefit ratio [1].

Hormonal contraceptives

Another therapy for PCOS supported by guidelines is an ovary-silencing approach and includes HCs having different combinations of the estro-progestins [8,14]. HCs are the first line therapy in menstrual abnormalities, hirsutism, and acne in PCOS [1]. There are ongoing studies to evaluate the efficacy or superiority of one form of HCs over another in patients with obesity and PCOS. Both weight loss and HCs result in significant improvement in several physical and mental parameters which are related to quality of life, depressive symptoms, and anxiety disorders [14].

Drospirenone as the progestogen source of HCs appears to be more beneficial for long-term cardiovascular and metabolic factors as compared to chlormadinone acetate as the progestogen source [15]. Drospirenone improves hyperandrogenism and insulin resistance. There have been favorable effects on lipid profiles and high sensitive C-reactive protein (hsCRP) levels [15]. Likewise, when different ethinylestradiol (EE) doses were compared in a study, it was found that along with drospirenone, 20μg EE results are as effective as 30μg EE in improving the clinical and hormonal features of normal weight PCOS women, while exhibiting a milder influence on metabolic parameters [16-17]. Both 20- and 30-μg regimens induced a significant improvement in hirsutism, testosterone, dehydroepiandrosterone sulfate (DHEAS), and SHBG levels. Both formulations did not significantly modify glucose-insulinemic metabolism. Triglyceride levels were increased with both the formulations, but more markedly in the 30-μg EE dose [17]. More studies are needed in obese PCOS patients to understand the effects of different HC components and doses on obesity and metabolic indices. Similarly, in a comparison between the new remedy PioFluMet and the traditional therapy (i.e., HCs) in adolescent girls with hyperandrogenemia, PioFluMet appears to be equally effective in reducing serum androgen levels and inflammatory indicators after one year of use. Furthermore, a low dose PioFluMet regimen is more effective in improving the metabolic and cardiovascular aberrations linked with PCOS and hyperandrogenism [8].   

Inositols                                     

In recent years, the introduction of inositols in the treatment plan of PCOS has proved to be very useful in dealing with the related endocrine-metabolic disorders. Treatment with two inositol isomers, myo-inositol and d-chiro-inositol, could be proposed as a favorable therapeutic approach for the treatment of patients with PCOS. Their physiological plasma ratio is 40:1. Inositols improve insulin resistance, serum androgen levels and many features of metabolic syndrome, thus also reducing cardiovascular risk [18-19].

Inositols belong to the sugar family and are an important part of structural lipids [20]. They are known to play a role in enzyme activation that controls glucose metabolism. Among them, myo-inositol is the most distributed in biological systems. Both prokaryotic and eukaryotic cells synthesize and incorporate it into the cell membrane [20]. Myo-inositol is the precursor of inositol triphosphate, a second messenger regulating many hormones such as thyroid-stimulating hormone (TSH), follicle-stimulating hormone (FSH), and insulin. D-chiro-inositol also provides second messengers that regulate glucose uptake and glycogen synthesis. As PCOS women are linked to insulin resistance, a defect in tissue availability or altered metabolism of inositols or inositol-phospho-glycan’s second messenger pathway may be contributing to insulin resistance [20]. Inositols improve the endocrine and metabolic parameters in young, overweight PCOS patients and regulate the menstrual cycle [18]. Treatment with inositols in obese PCOS patients is also effective in reducing BMI [21]. The decrease in BMI is without any lifestyle modification. D-chiro-inositols improve several endocrine parameters such as LH, LH/FSH, and androstenedione. Moreover, the myo-inositol administration is more effective in those obese patients who have high fasting insulin plasma levels or in those hyperinsulinemic PCOS patients who have a history of relatives with diabetes [21]. Myo-inositol has been more beneficial in improving the metabolic profile, whereas d-chiro-inositol reduces hyperandrogenism better than myo-inositol [22]. Inositols also have the potential to restore spontaneous ovulation and improve fertility in women with PCOS [23].

D-chiro-inositol alone, mostly when given at a high dosage, exerts an unfavorable effect on oocyte quality [19]. However, the combination therapy of myo-inositol along with d-chiro-inositol yields better clinical results; Nordio, et al. proposed a physiological plasma ratio of 40:1 [24]. A combination of myo-inositol and d-chiro-inositol counteract PCOS at both systemic and ovary levels [19]. These clinical trials highlight the beneficial effects of inositol supplementation in improving several of the hormonal and reproductive disturbances of PCOS as well as obesity linked with PCOS.

There is a proposed link between inositols, the new insulin-sensitizing molecule, and other compounds that can increase their therapeutic effect. One of these associations is with the monacolin K, a natural statin. It reduces cholesterol, androgens, and lipoic acid by a mechanism affecting steroidogenesis [25]. It is also known for its anti-inflammatory, antioxidant and insulin-sensitizing activity. Results have shown the good efficacy of myoinositol, lipoic acid, and monacolin K combinations. There was a significant improvement in lipid parameters and hyperandrogenism indices [25]. An association between myo-inositol and alpha-lipoic acid was established recently in women with PCOS. Alpha-lipoic acid is a powerful natural antioxidant and an enzyme cofactor of the mitochondrial respiratory chain [26]. It improves the glycemic control in patients with T2DM. Another study demonstrated that a combination of metformin, myo-inositol, and alpha-lipoic acid leads to an improvement in hyperandrogenism [26]. BMI and insulin resistance improves significantly as compared to treatment with metformin alone. This combination represents an excellent therapeutic choice for those obese women with PCOS who do not want to take HCs [26]. By reducing hyperinsulinemia, insulin-sensitizing agents may improve endocrine and reproductive abnormalities in obese PCOS patients [26]. Future clinical studies with a larger number of subjects are required to evaluate these effects more closely and to test their therapeutic implications.

Adiponectin

There is an association between PCOS and obesity with low adiponectin levels. Low adiponectin levels may be associated with insulin resistance. Adipose tissue hormones (adipokines), like adiponectin, have an important physiological role in limiting androgen production by the ovary [27]. Drugs are being developed that target adiponectin for the management of metabolic and cardiovascular dysfunction. These drugs may also provide therapeutic benefit for women with PCOS [27]. Moreover, adiponectin levels can likely be used as a biomarker even in lean young women with PCOS or in women with a family history of PCOS [28]. The mechanism of action, by which adiponectin exerts its influence on the metabolic indices and androgen excess in the overweight PCOS women, include probable reduction of adiponectin serum levels as well as a reduction in the expression of adiponectin receptors in theca cells of PCOS patients due to the defective action of adiponectin [27]. Bovine theca cells treated with adiponectin showed a significant reduction in gene expression in the key androgen synthesis enzymes. It is still not clear whether the changes in adiponectin receptors are because of a cause or a consequence of obesity or insulin resistance [27]. Quercetin is a flavonoid which increases the levels of adiponectin by 5.56% and is found to influence adiponectin-mediated insulin sensitivity in women with PCOS [29]. While rodent models have shown an improvement in PCOS phenotype after adiponectin (brown adipose tissue-induced) treatment, there is a lack of evidence in favor of its clinical use in this regard [30]. Today, adiponectin in research is favored more as a biomarker than as a treatment option in patients with PCOS. 

Orlistat

Anti-obesity drugs such as orlistat, sibutramine, and rimonabant have been studied in PCOS. The latter two agents were withdrawn because of their side effects [31]. In the case of orlistat, evidence is limited that it is beneficial for overweight women with PCOS. Several long-term studies on orlistat in overweight diabetic patients have shown significant weight loss and improved metabolic and cardiovascular indices as compared to pursuing lifestyle changes alone [31]. Orlistat use in obese PCOS patients leads to an improvement in insulin resistance, hyperandrogenemia, and cardiovascular risk factors [31]. Orlistat in combination with lifestyle modification (i.e., diet, exercise, and behavioral changes) exerts a useful effect on the endocrine and metabolic aberrations in obese patients with PCOS [31]. Apart from an improvement in hyperandrogenism and insulin sensitivity, orlistat supposedly increases serum anti-müllerian hormone (AMH) levels in obese PCOS patients [32]. The available evidence also indicates that orlistat and metformin have similar effects in reducing BMI, insulin resistance markers, and serum testosterone levels in obese women with PCOS [33-34]. Orlistat can induce ovulation and delivers similar ovulation rates as metformin in obese PCOS patients. Moreover, orlistat has minimal side-effects and is better tolerated as compared to metformin [34].  

Vitamin D and calcium

Vitamin D and calcium seem to have therapeutic implications in improving the hormonal and metabolic indices in PCOS. However, conflicting evidence exists in this regard. Serum 25-hydroxyvitamin D was improved in obese PCOS patients who were given daily vitamin D and elemental calcium. A significant reduction in total testosterone and androgens levels (by 12% and 17%, respectively) were seen after three months supplementation of 530mg elemental Ca with vitamin D 3533 IU later increased to 8533 IU [35]. Patient blood pressure profiles were also improved. Appropriately designed studies are needed to better explore the proposed benefits of vitamin D and calcium in the treatment of PCOS [35-36]. Serum AMH is elevated in women with PCOS and shows an abnormal ovarian folliculogenesis. Vitamin D therapy normalizes the serum AMH levels and, thereby, could be linked to an improvement in folliculogenesis [37]. On the contrary, a meta-analysis reviewing the role of vitamin D in PCOS found no evidence that vitamin D supplementation reduces metabolic and hormonal deregulations in PCOS [38]. Rather, vitamin D deficiency may be a comorbid manifestation of PCOS or a minor pathway in PCOS-linked hormonal and metabolic abnormalities. More trials of appropriate size are required. Another study shows that insulin sensitivity is unchanged with high-dose vitamin D treatment in PCOS, but there is a decrease in two-hour glucose and insulin levels after a 75-g oral glucose tolerance test [39]. Diastolic blood pressure profiles also improved [39].

Statins

There is contradictory evidence about the use of statins in PCOS. So far, routine administration of statins along with metformin has not been well established in women with PCOS. A Tibetan research center studied simvastatin and found that it improves endothelial functions and insulin resistance in PCOS patients after drug administration for six months; Homeostasis Model Assessment of Insulin Resistance (HOMA-IR), lipid profile, and BMI improved in these PCOS women [40]. Statins have shown some improvement in hyperandrogenism [41]. But their use is associated with impairment of glucose metabolism and an increased risk of T2DM. That is why statins should not be started for treating PCOS, but should only be started according to the accepted criteria for cardiovascular disease risk assessments [41]. In a study to explore the effects of atorvastatin therapy on hormonal and metabolic parameters in women with PCOS, lipid profile and inflammatory markers showed improvement, but insulin sensitivity worsened during six months of atorvastatin therapy (20mg) [41]. Serum levels of DHEAS decreased, whereas no change was observed in serum testosterone levels, perhaps because the study group had women who were relatively old and had normal testosterone levels [41]. Another meta-analysis to investigate the effects of combined statins and metformin regimens on PCOS showed similar results [42]. Combined statins and metformin therapy can improve lipid and inflammation parameters, but cannot effectively improve insulin sensitivity and hyperandrogenism in women with PCOS, when compared to metformin alone [42]. A large-scale randomized controlled study must be conducted to ascertain the long-term effects of the therapy [42]. The Endocrine Society clinical practice guidelines disapprove statins for the time being for use in PCOS and recommend further studies [1].

Bariatric surgery

Bariatric surgery is recommended in morbidly obese women with PCOS or obese women with metabolic comorbidities. Morbidly obese women with PCOS, who have undergone Roux-en-Y gastric bypass surgery have shown improvement in cardio-metabolic disease risk at one year after the procedure [43]. Decreases in BMI, glycosylated hemoglobin (HbA1c), total cholesterol, LDL, and triglyceride levels were also seen [43]. 

Non-pharmacological treatment

Various studies have been done in China to suggest the role of berberine (BBR) in the improvement of some of the metabolic and hormonal imbalances in PCOS. BBR is the major active element of Rhizoma coptidis and has shown positive effects on T2DM and insulin resistance [44]. It has also shown useful effects on lipid metabolism and metabolic syndrome [44]. Because BBR was previously found to improve insulin resistance in a way similar to metformin, BBR may also have the same regulatory effects on androgen synthesis as metformin in women with PCOS [44]. Multicenter studies in China are ongoing to further evaluate the effects of BBR on insulin resistance in women with PCOS [44]. BBR is effective and safe in the treatment of T2DM and dyslipidemia [45]. It also appears to have a greater effect on the changes in body composition and causes a reduction in the androgen concentration [46]. Furthermore, the waist-to-hip ratio decreases significantly in women treated with BBR as compared to those treated with metformin. This decrease in the waist-to-hip ratio with BBR treatment happens by inducing adipose tissue redistribution in the absence of an overall weight change. More studies are required to assess the favorable metabolic effects of BBR in obesity caused by PCOS [46]. BRB adverse effects include constipation.

Acupuncture is an ancient therapeutic approach in which needles are used for sensory stimulation of somatic afferent nerves that innervate skin and muscles [47]. In studies in China, acupuncture is said to regulate the hypothalamic-pituitary-ovarian axis, hence, it may be of relevance in improving hormonal and metabolic profile in hyperandrogenism [47].

Lately, vinegar has been found to be beneficial in improving insulin resistance in patients who wanted a non-pharmacological remedy for PCOS [48]. The patients took fifteen grams of apple vinegar every day for a period of 90 to 110 days. Vinegar intake resulted in a decrease of HOMA-IR as well as a decrease in the LH/FSH ratio. Vinegar possibly restores ovulatory functions and improves insulin sensitivity in PCOS patients [48].

Resveratrol, a polyphenol (found in red grape skin, nuts, and berries in response to injuries) significantly reduces ovarian and adrenal androgens [36]. This effect could be due to an improvement in insulin sensitivity and a decrease in insulin levels. Resveratrol treatment leads to a significant decrease in total testosterone by 23.1% and a 22.2% decrease in DHEAS [47,49]. Also, there is a decrease in fasting insulin level by 31.8% and an increase in the insulin sensitivity (66.3%) [49]. In a rodent model, resveratrol was found to reduce proliferation of theca cells. It also reduces androgen production by arresting Cyp17a1 messenger RNA gene expression. These findings may be of potential clinical relevance for conditions associated with excessive androgen production, such as PCOS [50]. The overall body of evidence in non-pharmacological treatments is weak and needs further evaluation and studies.

## Conclusions

Obesity and metabolic syndrome in PCOS are linked with insulin resistance and other metabolic aberrations associated with a higher risk of cardiovascular events. Several studies suggest the use of new or modified therapies for the treatment of obesity and metabolic syndrome associated with PCOS. Recent clinical trials have focused on inositols, statins, vitamin D, and quercetin for the treatment of obese women with PCOS. Non-pharmacological therapies are also under study. Its use as a combination or stand-alone therapy in women with PCOS needs to be investigated in future studies. Nonetheless, several pressing concerns remain to be addressed about these remedies including their efficacy and safety profiles. More research is needed to assign better therapeutic regimens in obese PCOS patients and strategies to prevent the development of comorbidities.

## References

[1] Legro RS, Arslanian SA, Ehrmann DA (2013). Diagnosis and treatment of polycystic ovary syndrome: an endocrine society clinical practice guideline. J Clin Endocrinol Metab.

[2] Azziz R, Carmina E, Dewailly D (2009). The Androgen Excess and PCOS Society criteria for the polycystic ovary syndrome: the complete task force report. Fertil Steril.

[3] Hart R, Doherty DA (2015). The potential implications of a pcos diagnosis on a woman’s long-term health using data linkage. J Clin Endocrinol Metab.

[4] Goodman NF, Cobin RH, Futterweit W (2017). American association of clinical endocrinologists, American college of endocrinology, and androgen excess and pcos society disease state clinical review: guide to the best practices in the evaluation and treatment of polycystic ovary syndrome - part 2. Endocr Pract.

[5] Vilmann LS, Thisted E, Baker JL, Holm JC (2012). Development of obesity and polycystic ovary syndrome in adolescents. Horm Res Paediatr.

[6] Diane A, Kupreeva M, Borthwick F, Proctor SD, Pierce WD, Vine DF (2015). Cardiometabolic and reproductive benefits of early dietary energy restriction and voluntary exercise in an obese PCOS-prone rodent model. J Endocrinol.

[7] Legro RS, Dodson WC, Kunselman AR (2016). Benefit of delayed fertility therapy with preconception weight loss over immediate therapy in obese women with pcos. J Clin Endocrinol Metab.

[8] Díaz M, Chacón MR, López-Bermejo A (2012). Ethinyl estradiol-cyproterone acetate versus low-dose pioglitazone-flutamide-metformin for adolescent girls with androgen excess: divergent effects on CD163, TWEAK receptor, ANGPTL4, and LEPTIN expression in subcutaneous adipose tissue. J Clin Endocrinol Metab.

[9] Kurzthaler D, Hadziomerovic-Pekic D, Wildt L, Seeber BE (2014). Metformin induces a prompt decrease in LH-stimulated testosterone response in women with pcos independent of its insulin-sensitizing effects. Reprod Biol Endocrinol.

[10] Codner E, Iñíguez G, López P (2013). Metformin for the treatment of hyperandrogenism in adolescents with type 1 diabetes mellitus. Horm Res Paediatr.

[11] Naderpoor N, Shorakae S, de Courten B, Misso ML, Moran LJ, Teede HJ (2015). Metformin and lifestyle modification in polycystic ovary syndrome: systematic review and meta-analysis. Hum Reprod Update.

[12] Stabile G, Borrielli I, Artenisio AC (2014). Effects of the insulin sensitizer pioglitazone on menstrual irregularity, insulin resistance and hyperandrogenism in young women with polycystic ovary syndrome. J Pediatr Adolesc Gynecol.

[13] Shahebrahimi K, Jalilian N, Bazgir N, Rezaei M (2016). Comparison clinical and metabolic effects of metformin and pioglitazone in polycystic ovary syndrome. Indian J Endocrinol Metab.

[14] Dokras A, Sarwer DB, Allison KC (2016). Weight loss and lowering androgens predict improvements in health-related quality of life in women with PCOS. J Clin Endocrinol Metab.

[15] Yildizhan R, Gokce AI, Yildizhan B, Cim N (2015). Comparison of the effects of chlormadinone acetate versus drospirenone containing oral contraceptives on metabolic and hormonal parameters in women with PCOS for a period of two-year follow-up. Gynecol Endocrinol.

[16] Bhattacharya SM, Jha A, DasMukhopadhyay L (2016). Comparison of two contraceptive pills containing drospirenone and 20 μg or 30 μg ethinyl estradiol for polycystic ovary syndrome. Int J Gynaecol Obstet.

[17] Romualdi D, De Cicco S, Busacca M, Gagliano D, Lanzone A, Guido M (2013). Clinical efficacy and metabolic impact of two different dosages of ethinyl-estradiol in association with drospirenone in normal-weight women with polycystic ovary syndrome: a randomized study. J Endocrinol Invest.

[18] Formuso C, Stracquadanio M, Ciotta L (2015). Myo-inositol vs. d-chiro inositol in pcos treatment. Minerva Ginecol.

[19] Dinicola S, Chiu TTY, Unfer V, Carlomagno G, Bizzarri M (2014). The rationale of the myo-inositol and d-chiro-inositol combined treatment for polycystic ovary syndrome. J Clin Pharmacol.

[20] Bizzarri M, Carlomagno G (2017). Inositol: history of an effective therapy for polycystic ovary syndrome. Eur Rev Med Pharmacol Sci.

[21] Genazzani AD, Santagni S, Rattighieri E (2014). Modulatory role of d-chiro-inositol (DCI) on LH and insulin secretion in obese pcos patients. Gynecol Endocrinol.

[22] Pizzo A, Laganà AS, Barbaro L (2014). Comparison between effects of myo-inositol and d-chiro-inositol on ovarian function and metabolic factors in women with PCOS. Gynecol Endocrinol.

[23] Unfer V, Nestler JE, Kamenov ZA, Prapas N, Facchinetti F (2016). Effects of inositol(s) in women with pcos: a systematic review of randomized controlled trials. Int J Endocrinol.

[24] Nordio M, Proietti E (2017). The combined therapy with myo-inositol and d-chiro-inositol reduces the risk of metabolic disease in PCOS overweight patients compared to myo-inositol supplementation alone. Eur Rev Med Pharmacol Sci.

[25] Morgante G, Cappelli V, Di Sabatino A, Massaro MG, De Leo V (2017). Polycystic ovary syndrome (PCOS) and hyperandrogenism: the role of a new natural association. Minerva Ginecol.

[26] Cappelli V, Di Sabatino A, Musacchio MC, De Leo V (2017). Evaluation of a new association between insulin-sensitizers and α-lipoic acid in obese women affected by PCOS (Article in Italian). Minerva Ginecol.

[27] Comim FV, Hardy K, Franks S (2013). Adiponectin and its receptors in the ovary: further evidence for a link between obesity and hyperandrogenism in polycystic ovary syndrome. PloS One.

[28] Mirza SS, Shafique K, Shaikh AR, Khan NA, Anwar Qureshi M (2014). Association between circulating adiponectin levels and polycystic ovarian syndrome. J Ovarian Res.

[29] Rezvan N, Moini A, Janani L (2016). Effects of quercetin on adiponectin-mediated insulin sensitivity in polycystic ovary syndrome: a randomized placebo-controlled double-blind clinical trial. Horm Metab Res.

[30] Yuan X, Hu T, Zhao H (2016). Brown adipose tissue transplantation ameliorates polycystic ovary syndrome. Proc Natl Acad Sci U S A.

[31] Panidis D, Tziomalos K, Papadakis E, Vosnakis C, Chatzis P, Katsikis I (2013). Lifestyle intervention and anti-obesity therapies in the polycystic ovary syndrome: impact on metabolism and fertility. Endocrine.

[32] Vosnakis C, Georgopoulos NA, Rousso D (2013). Diet, physical exercise and orlistat administration increase serum anti-müllerian hormone (AMH) levels in women with polycystic ovary syndrome (PCOS). Gynecol Endocrinol.

[33] Graff SK, Mario FM, Ziegelmann P, Spritzer PM (2016). Effects of orlistat vs. metformin on weight loss-related clinical variables in women with PCOS: systematic review and meta-analysis. Int J Clin Pract.

[34] Kumar P, Arora S (2014). Orlistat in polycystic ovarian syndrome reduces weight with improvement in lipid profile and pregnancy rates. J Hum Reprod Sci.

[35] Pal L, Berry A, Coraluzzi L, Kustan E, Danton C, Shaw J, Taylor H (2012). Therapeutic Implications of vitamin D and calcium in overweight women with polycystic ovary syndrome. Gynecol Endocrinol.

[36] Mansour A, Hosseini S, Larijani B, Mohajeri-Tehrani MR (2016). Nutrients as novel therapeutic approaches for metabolic disturbances in polycystic ovary syndrome. EXCLI J.

[37] Irani M, Minkoff H, Seifer DB, Merhi Z (2014). Vitamin D increases serum levels of the soluble receptor for advanced glycation end products in women with PCOS. J Clin Endocrinol Metab.

[38] He C, Lin Z, Robb SW, Ezeamama AE (2015). Serum vitamin D levels and polycystic ovary syndrome: a systematic review and meta-analysis. Nutrients.

[39] Raja-Khan N, Shah J, Stetter CM, Lott ME, Kunselman AR, Dodson WC, Legro RS (2014). High-dose vitamin D supplementation and measures of insulin sensitivity in polycystic ovary syndrome: a randomized controlled pilot trial. Fertil Steril.

[40] Yang B, Sun Z-J, Chen B (2017). Statin ameliorates endothelial dysfunction and insulin resistance in Tibet women with polycystic ovary syndrome. Eur Rev Med Pharmacol Sci.

[41] Puurunen J, Piltonen T, Puukka K (2013). Statin therapy worsens insulin sensitivity in women with polycystic ovary syndrome (PCOS): a prospective, randomized, double-blind, placebo-controlled study. J Clin Endocrinol Metab.

[42] Sun J, Yuan Y, Cai R (2015). An investigation into the therapeutic effects of statins with metformin on polycystic ovary syndrome: a meta-analysis of randomised controlled trials. BMJ Open.

[43] Gomez-Meade CA, Lopez-Mitnik G, Messiah SE, Arheart KL, Carrillo A, de la Cruz-Muñoz N (2013). Cardiometabolic health among gastric bypass surgery patients with polycystic ovarian syndrome. World J Diabetes.

[44] Li Y, Ma H, Zhang Y, Kuang H, Ng EH, Hou L, Wu X (2013). Effect of berberine on insulin resistance in women with polycystic ovary syndrome: study protocol for a randomized multicenter controlled trial. Trials.

[45] Zhang Y, Li X, Zou D (2008). Treatment of type 2 diabetes and dyslipidemia with the natural plant alkaloid berberine. J Clin Endocrinol Metab.

[46] Wei W, Zhao H, Wang A (2012). A clinical study on the short-term effect of berberine in comparison to metformin on the metabolic characteristics of women with polycystic ovary syndrome. Eur J Endocrinol.

[47] Aquino CI, Nori SL (2017). Complementary therapy in polycystic ovary syndrome. Transl Med UniSa.

[48] Wu D, Kimura F, Takashima A (2017). Intake of vinegar beverage is associated with restoration of ovulatory function in women with polycystic ovary syndrome. Tohoku J Exp Med.

[49] Banaszewska B, Wrotyńska-Barczyńska J, Spaczynski RZ, Pawelczyk L, Duleba AJ (2016). Effects of resveratrol on polycystic ovary syndrome: a double-blind, randomized, placebo-controlled trial. J Clin Endocrinol Metab.

[50] Ortega I, Villanueva JA, Wong DH, Cress AB, Sokalska A, Stanley SD, Duleba AJ (2012). Resveratrol reduces steroidogenesis in rat ovarian theca-interstitial cells: the role of inhibition of Akt/PKB signaling pathway. Endocrinology.

